# Supplementary material for: Early post-infection treatment of SARS-CoV-2 infected macaques with human convalescent plasma with high neutralizing activity reduces lung inflammation
Source: bioRxiv. 2021 Sep 1:2021.09.01.458520. Preprint. [Version 1] doi: 10.1101/2021.09.01.458520 (PMC8423222; doi:10.1101/2021.09.01.458520)
Supplement: 1 [file NIHPP2021.09.01.458520V1-supplement-1.pdf]

SUPPLEMENT

SARS-COV-2 antigen reactivity

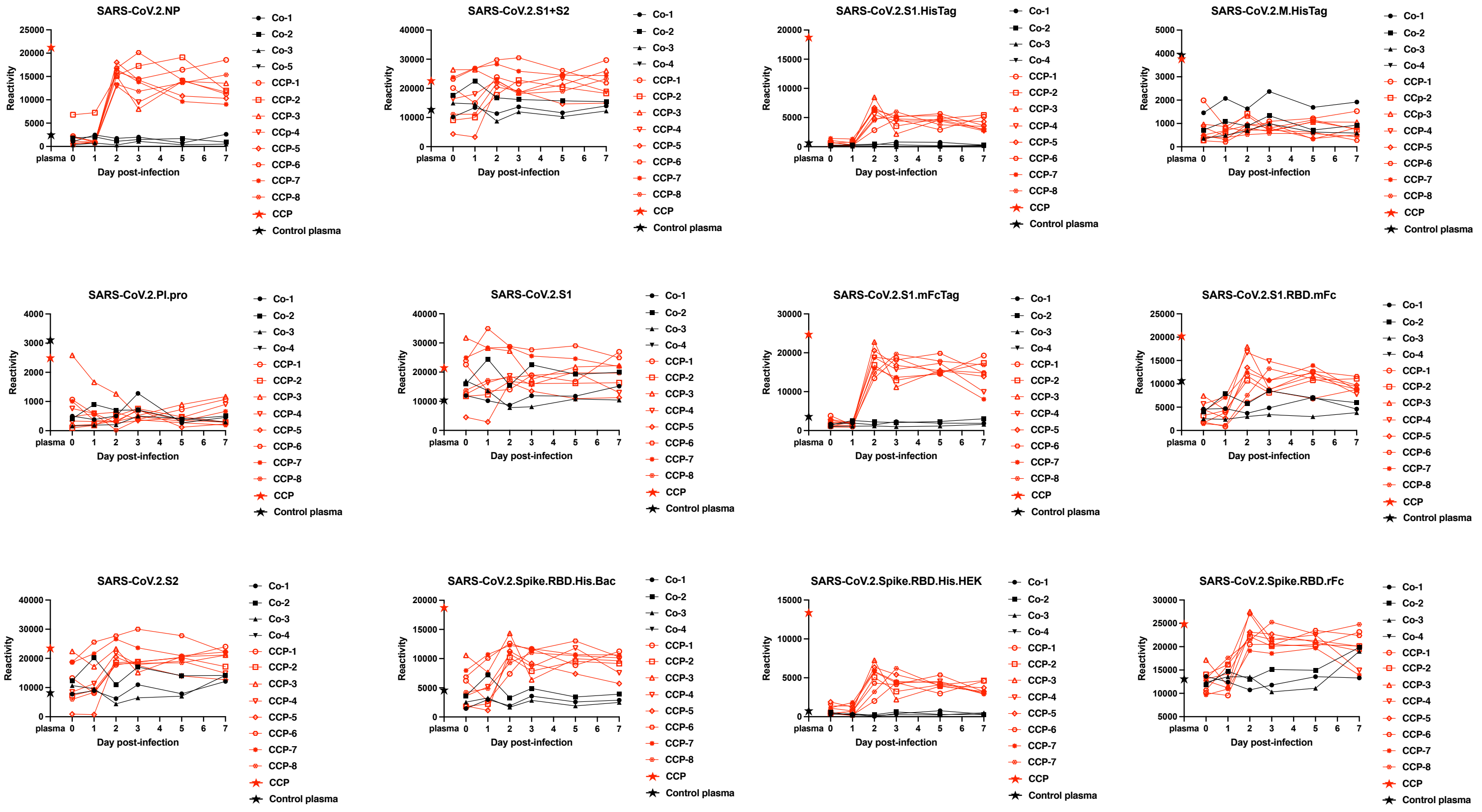

Figure S1: Reactivity of macaque plasma to SARS-CoV-2 antigens following human plasma infusion.
Plasma collected of the macaques before and after infusion with pooled CCP or normal control plasma was tested by coronavirus antigen microarray assay (COVAM). The data on reactivity to SARS-CoV-2 antigens in this assay are represented as individual graphs. The reactivity of the CCP and normal plasma (see **Fig. 1A**) is indicated on the Y-axis as red and black stars, respectively, to demonstrate the dilution effect after transfusion into the macaques.

bioRxiv preprint doi: https://doi.org/10.1101/2021.09.01.458520; this version posted September 1, 2021. The copyright holder for this preprint (which was not certified by peer review) is the author/funder. This article is a US Government work. It is not subject to copyright under 17 USC 105 and is also made available for use under a CC0 license.

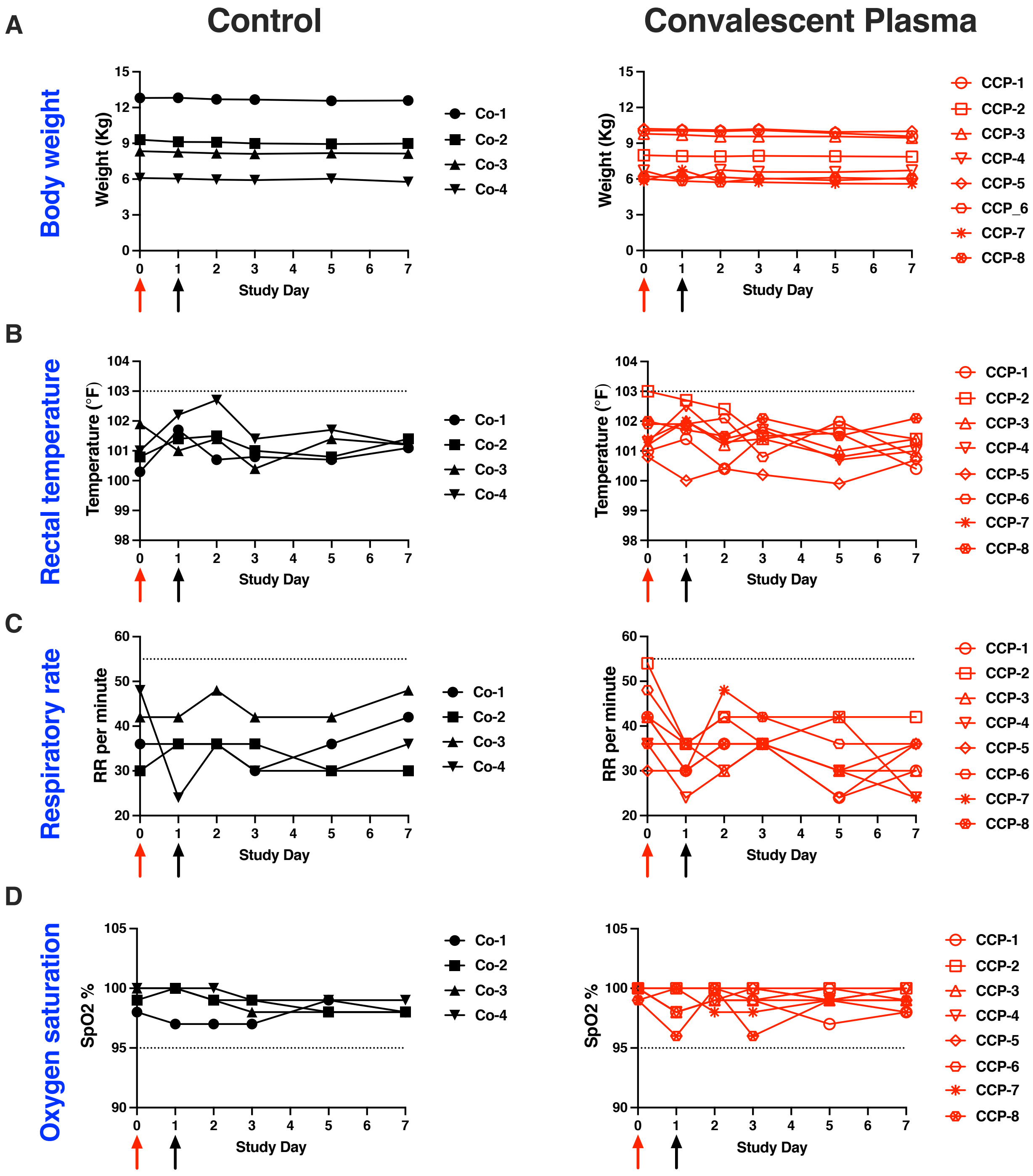

Fig. S2. Clinical measurements collected at time of sedation.

Red and black arrows indicate time of virus inoculation and monoclonal antibody administration on days 0 and 1, respectively. (A) Body weight remained stable. (B) Rectal temperature; horizontal line indicates the cut-off of 103° F, above which ketoprofen treatment was administered. (C) Respiratory rate; the horizontal line indicates a cut-off value of 55 (per minute) as upper normal range. (D) Oxygen saturation measured by pulse oximetry; the horizontal line

bioRxiv preprint doi: https://doi.org/10.1101/2021.09.01.458520; this version posted September 1, 2021. The copyright holder for this preprint (which was not certified by peer review) is the author/funder. This article is a US Government work. It is not subject to copyright under 17 USC 105 and is also made available for use under a CC0 license.

indicates 95% as the lower end cut-off of the normal range. Total clinical scores, including the markers not graphed above, are presented in Fig. 4.

bioRxiv preprint doi: https://doi.org/10.1101/2021.09.01.458520; this version posted September 1, 2021. The copyright holder for this preprint (which was not certified by peer review) is the author/funder. This article is a US Government work. It is not subject to copyright under 17 USC 105 and is also made available for use under a CC0 license.

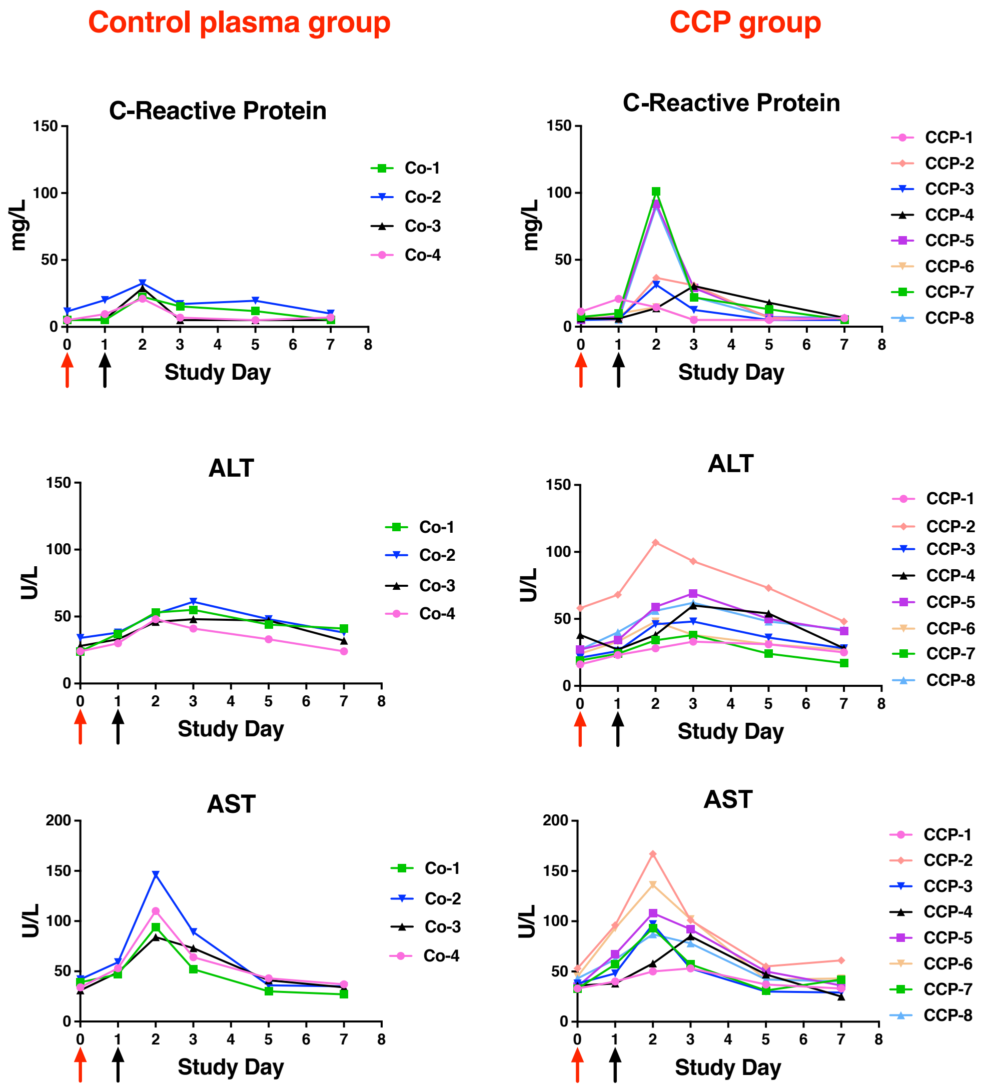

Fig. S3. Time course of serum chemistry markers in SARS-CoV-2 inoculated animals. Biochemistry analysis on serum samples was performed using Piccolo® BioChemistry Plus disks. (A) through (C) present C-reactive protein (CRP), alanine aminotransferase (ALT), and aspartate aminotransferase (AST), which showed transient changes during the early stages of infection regardless of the study group. Other markers in the panel did not show any obvious changes. Red and black arrows indicate time of virus inoculation and plasma administration on days 0 and 1, respectively.

bioRxiv preprint doi: https://doi.org/10.1101/2021.09.01.458520; this version posted September 1, 2021. The copyright holder for this preprint (which was not certified by peer review) is the author/funder. This article is a US Government work. It is not subject to copyright under 17 USC 105 and is also made available for use under a CC0 license.

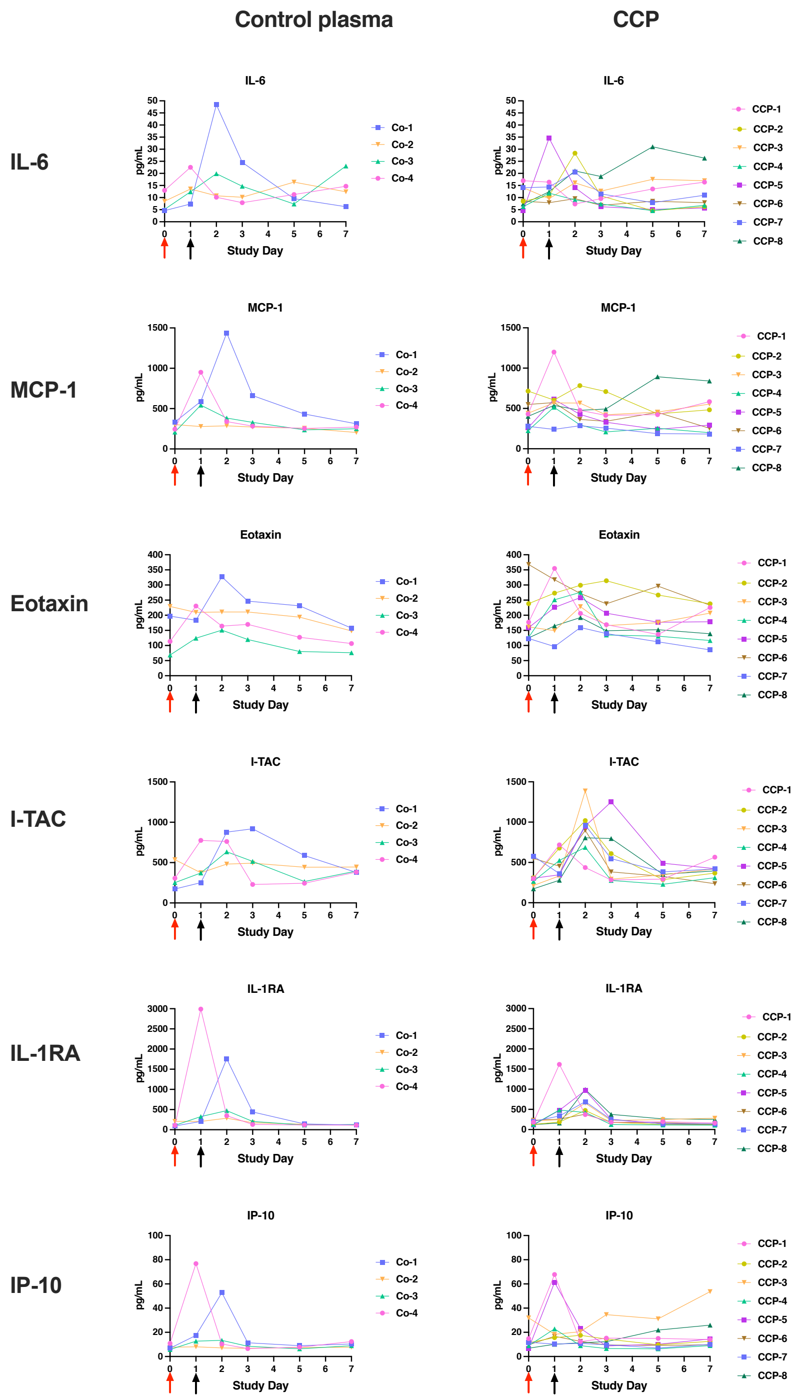

Fig. S4. Time course of cytokines and chemokines in plasma of SARS-CoV-2 inoculated animals.

Cytokines and chemokines were measured in plasma using established Luminex-based methodology (see methods section). Red and black arrows indicate time of virus inoculation and plasma administration on days 0 and 1, respectively. Markers on this figure represent ones that showed the most visible changes after infection. For other markers, see **Fig. S5**.

bioRxiv preprint doi: https://doi.org/10.1101/2021.09.01.458520; this version posted September 1, 2021. The copyright holder for this preprint (which was not certified by peer review) is the author/funder. This article is a US Government work. It is not subject to copyright under 17 USC 105 and is also made available for use under a CC0 license.

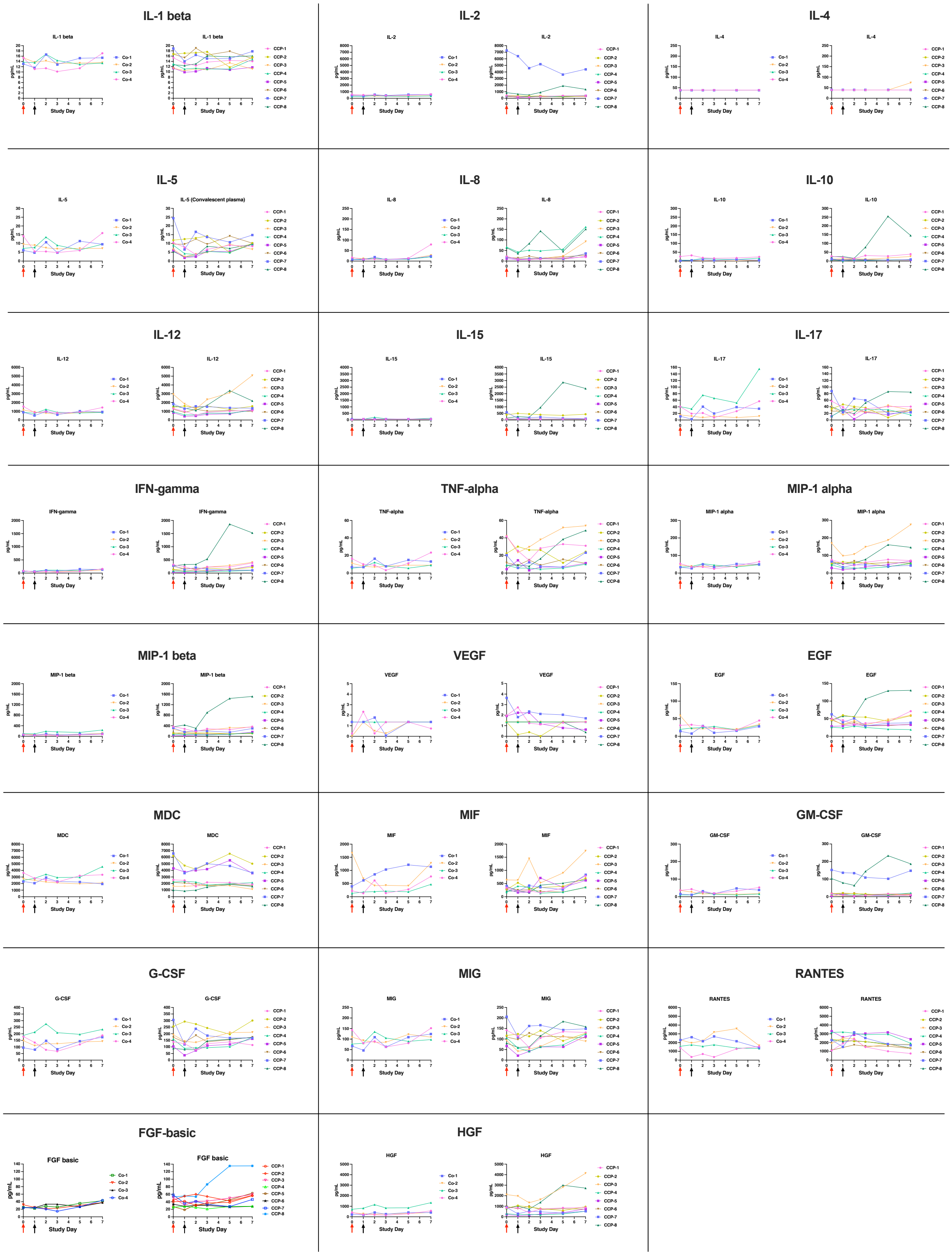

Fig. S5. Time course of additional cytokines and chemokines in plasma of SARS-CoV-2 inoculated animals.

Cytokines and chemokines presented in this panel were ones that did not show consistent changes among animals. The legend is the same as that of **Fig. S4**.

bioRxiv preprint doi: https://doi.org/10.1101/2021.09.01.458520; this version posted September 1, 2021. The copyright holder for this preprint (which was not certified by peer review) is the author/funder. This article is a US Government work. It is not subject to copyright under 17 USC 105 and is also made available for use under a CC0 license.

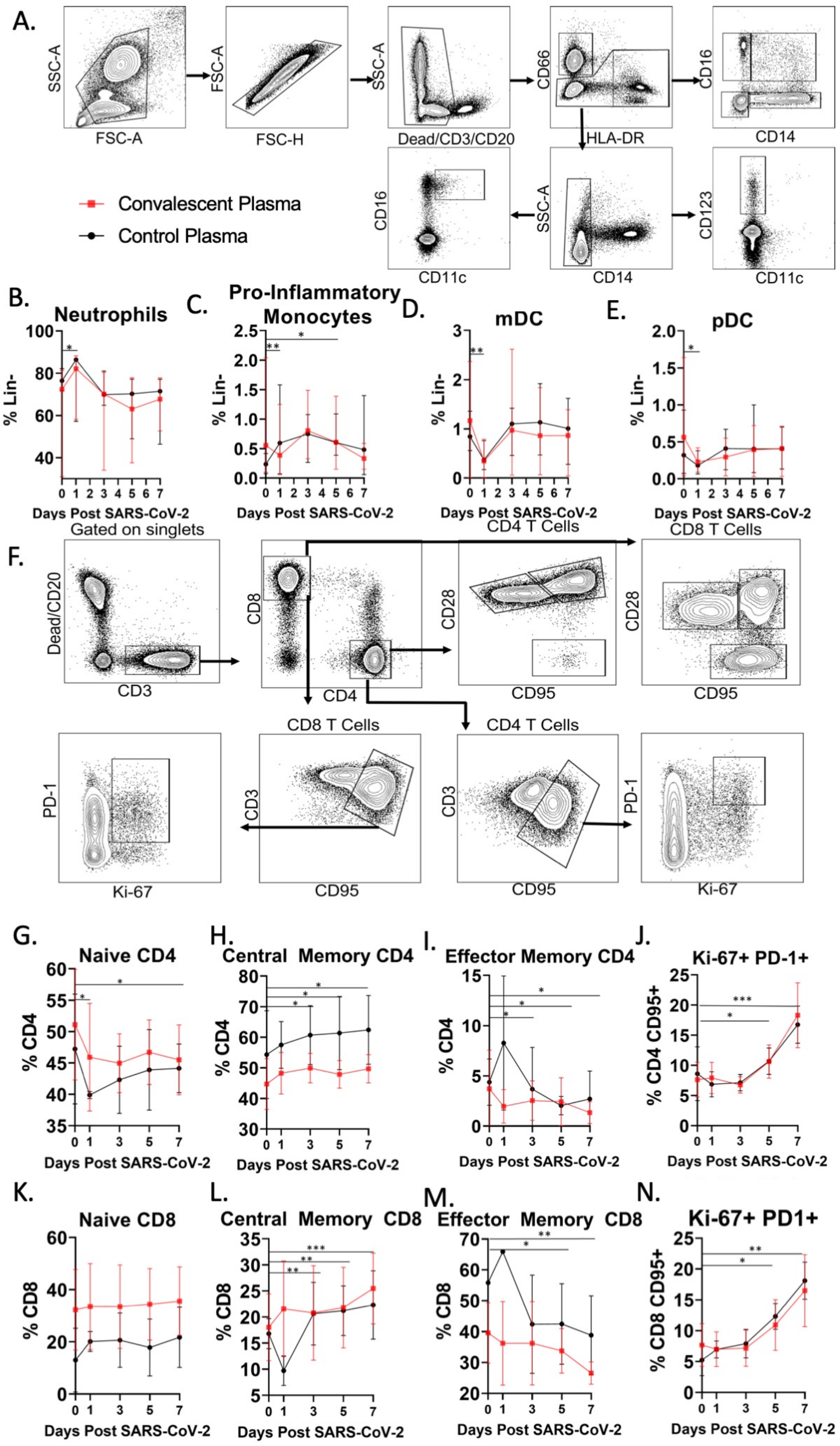

Fig. S6. Innate and adaptive immune responses following infection

(A) Representative gating strategy for innate immune cells in whole blood. Fluorochromes used: CD66:APC, CD20/CD3/Dead: APC-Cy7, ,Ki67:AF488,

bioRxiv preprint doi: https://doi.org/10.1101/2021.09.01.458520; this version posted September 1, 2021. The copyright holder for this preprint (which was not certified by peer review) is the author/funder. This article is a US Government work. It is not subject to copyright under 17 USC 105 and is also made available for use under a CC0 license.

CD14:AF700, CD123:BV421, CD16:BV605, HLA-DR:BV786, CD11c:PE-Cy7. Kinetics of circulating neutrophils, proinflammatory monocytes, mDCs, and pDCs measured at 0,1,3,5, and 7 days post SARS-CoV-2 infection. (B) Representative T cell gating strategy from whole blood. Fluorochromes used: CD25: APC, CD20/Dead: APC-Cy7, Ki67:AF488, CD3:AF700, CD95:BUV737, CD8:BUV805, CD4:BV650, CD69:BV711, CD28:PECF594, PD-1:PE-Cy7. Kinetics of circulating naïve, central memory, effector memory populations, and Ki67+PD-1+ memory cells of CD4 T cell. Kinetics of circulating naïve, central memory, effector memory, and CD69+ effector memory CD8 T cells. Significance was calculated using one tailed paired t test comparing pooled convalescent plasma and normal plasma animals against Day 0 *p=0.05, **p=0.01, ***p=0.001. Statistical analysis yielded no significant different between convalescent and normal plasma groups.

bioRxiv preprint doi: https://doi.org/10.1101/2021.09.01.458520; this version posted September 1, 2021. The copyright holder for this preprint (which was not certified by peer review) is the author/funder. This article is a US Government work. It is not subject to copyright under 17 USC 105 and is also made available for use under a CC0 license.

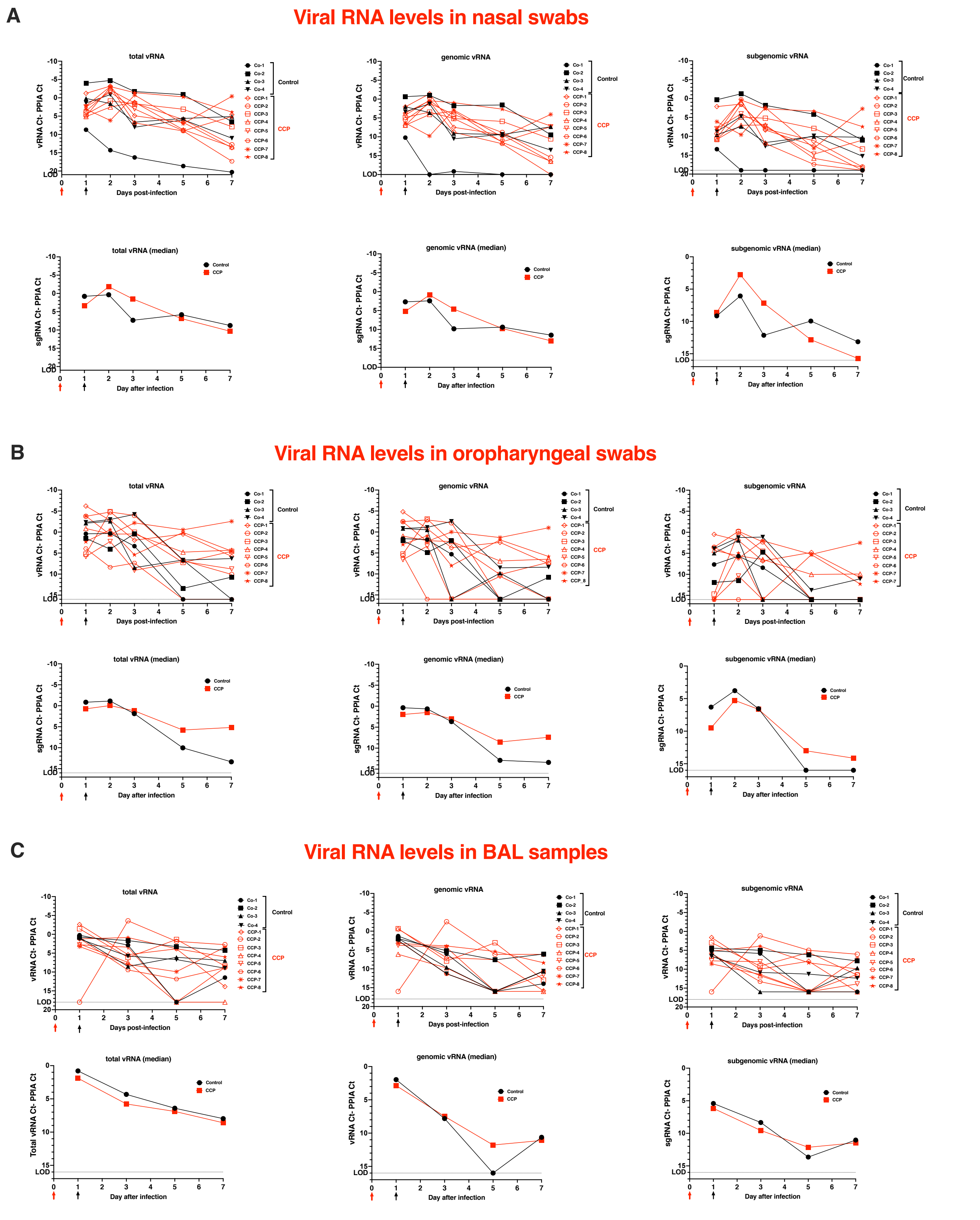

Fig. S7. Viral RNA levels in nasal swabs, oropharyngeal swabs and BAL samples.

bioRxiv preprint doi: https://doi.org/10.1101/2021.09.01.458520; this version posted September 1, 2021. The copyright holder for this preprint (which was not certified by peer review) is the author/funder. This article is a US Government work. It is not subject to copyright under 17 USC 105 and is also made available for use under a CC0 license.

Nasal swabs (A), oropharyngeal swabs (B) and BAL (cell pellets with supernatant) (C) were tested by RT-qPCR for total, genomic and subgenomic viral RNA, and the housekeeping gene PPIA mRNA. Viral RNA levels are expressed relative to PPIA mRNA by graphing the difference in Ct values. For each sample type, the top figures show the individual data (with the intersection of X-axis and Y-axis set near the limit of detection); the bottom figures display the median values per group. Red and black arrows indicate time of virus inoculation and monoclonal antibody administration on days 0 and 1, respectively.

bioRxiv preprint doi: https://doi.org/10.1101/2021.09.01.458520; this version posted September 1, 2021. The copyright holder for this preprint (which was not certified by peer review) is the author/funder. This article is a US Government work. It is not subject to copyright under 17 USC 105 and is also made available for use under a CC0 license.

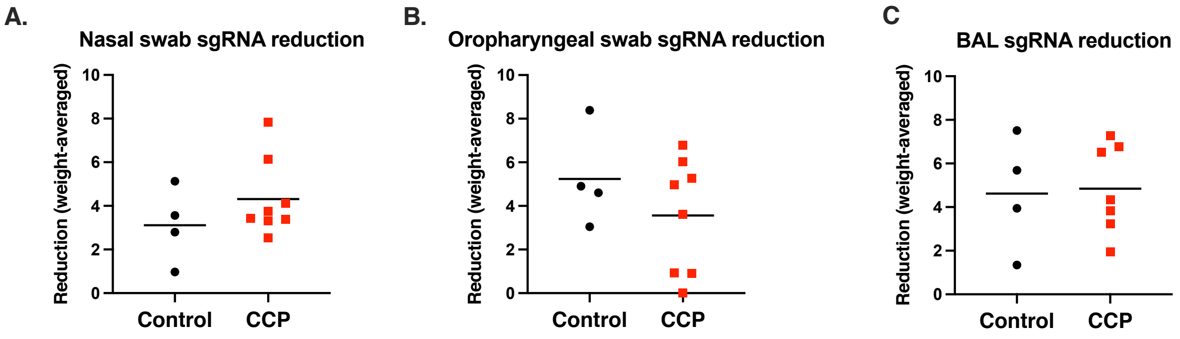

Fig. S8. **Lack of effect of convalescent plasma on sgRNA kinetics in nasal and oropharyngeal swabs and BAL of SARS-CoV-2 infected macaques.**

A weighted average analysis was performed on the sgRNA data from nasal and oropharyngeal swabs and BAL (**Fig. S7**) to calculate the relative decline of viral RNA (relative to cellular mRNA in the sample) from day 1 to day 7. For each animal, the AUC of relative sgRNA per cellular mRNA over time was tabulated using day 1 as baseline value, and then divided by 6 days to get the weighted average in the decline of sgRNA over the 6-day time period. Lines indicate mean values. On panel C, animal CCP-2 was excluded, as it had no detectable viral RNA in the BAL sample, which precluded this analysis. Statistical analysis revealed no effects between the control and CCP groups (panel A, p=0.29; panel B: p=0.30; panel C, p=0.88; unpaired t-test).

bioRxiv preprint doi: https://doi.org/10.1101/2021.09.01.458520; this version posted September 1, 2021. The copyright holder for this preprint (which was not certified by peer review) is the author/funder. This article is a US Government work. It is not subject to copyright under 17 USC 105 and is also made available for use under a CC0 license.

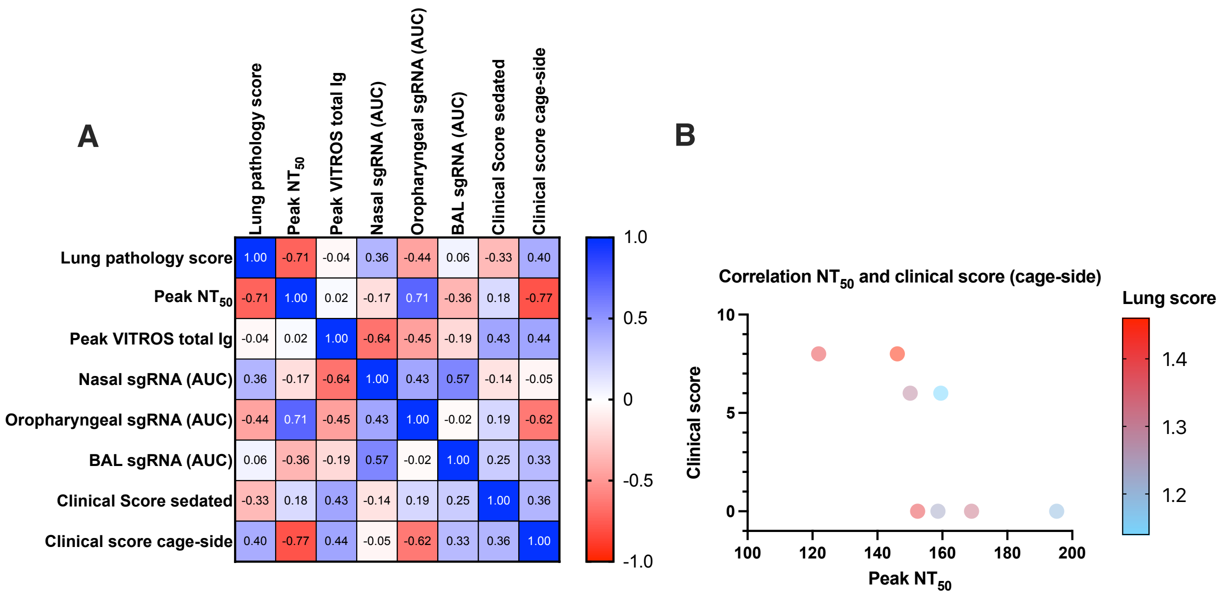

	Lung pathology score	Peak NT_{50}	Peak VITROS total Ig	Nasal sgRNA (AUC)	Oropharyngeal sgRNA (AUC)	BAL sgRNA (AUC)	Clinical Score sedated	Clinical score cage-side
Lung pathology score	1.00	-0.71	-0.04	0.36	-0.44	0.06	-0.33	0.40
Peak NT_{50}	-0.71	1.00	0.02	-0.17	0.71	-0.36	0.18	-0.77
Peak VITROS total Ig	-0.04	0.02	1.00	-0.64	-0.45	-0.19	0.43	0.44
Nasal sgRNA (AUC)	0.36	-0.17	-0.64	1.00	0.43	0.57	-0.14	-0.05
Oropharyngeal sgRNA (AUC)	-0.44	0.71	-0.45	0.43	1.00	-0.02	0.19	-0.62
BAL sgRNA (AUC)	0.06	-0.36	-0.19	0.57	-0.02	1.00	0.25	0.33
Clinical Score sedated	-0.33	0.18	0.43	-0.14	0.19	0.25	1.00	0.36
Clinical score cage-side	0.40	-0.77	0.44	-0.05	-0.62	0.33	0.36	1.00

Fig. S9. Multivariable correlation analysis on CCP-treated animals.

Multivariate analysis was performed on the 8 CCP-treated animals only. (A). Spearman r correlation matrix in heatmap format. For this analysis, the markers used are the same ones as in Fig. 7. (B) Correlation between neutralizing antibody peak NT_{50} values and clinical scores based on cage-side observations (Spearman r = -0.77; p=0.04). The labels next to each symbol indicate the individual animals.

bioRxiv preprint doi: https://doi.org/10.1101/2021.09.01.458520; this version posted September 1, 2021. The copyright holder for this preprint (which was not certified by peer review) is the author/funder. This article is a US Government work. It is not subject to copyright under 17 USC 105 and is also made available for use under a CC0 license.

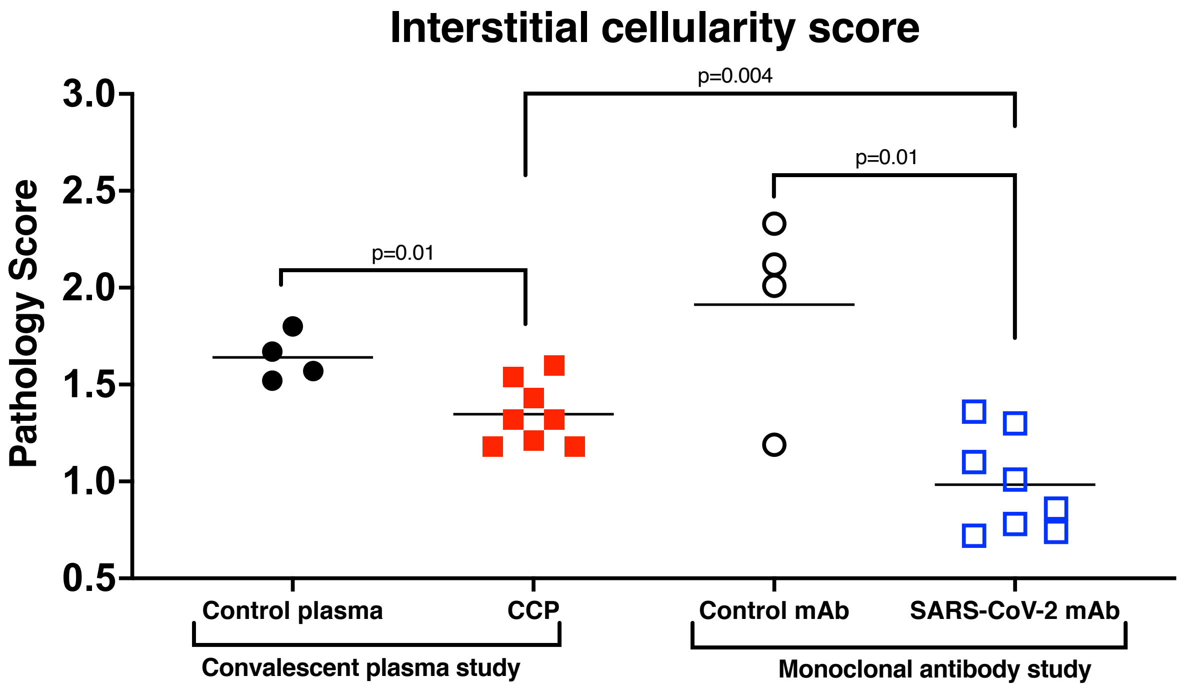

Fig. S10: Comparison of effects of convalescent plasma and monoclonal antibodies on reducing lung inflammation in SARS-CoV-2 infected macaques.

In an earlier study that used the same experimental procedures, we demonstrated that a combination of 2 potent anti-SARS-CoV-2 monoclonal antibodies (mAb; C135-LS and C144-LS) administered one day after virus inoculation reduced lung interstitial cellularity scores in 8 treated animals comparison to 4 animals treated with a control mAb [27]. Comparison of the CCP-treated and SARS-CoV-2 mAb-treated groups demonstrated that mAbs are more effective than CCP in reducing interstitial cellularity scores (p=0.004, unpaired t test).

Because in the monoclonal antibody study, scores were based on 3 lung lobes, the data of the CCP study presented in this figure are tabulated based on those same 3 lung lobes; using the data of all 7 lung lobes on the current CCP study (presented in **Fig. 5**) resulted in the same conclusions.

bioRxiv preprint doi: https://doi.org/10.1101/2021.09.01.458520; this version posted September 1, 2021. The copyright holder for this preprint (which was not certified by peer review) is the author/funder. This article is a US Government work. It is not subject to copyright under 17 USC 105 and is also made available for use under a CC0 license.

Table S1: Preparation of pooled COVID convalescent plasma.

Truncated DIN	% of total pool	NT_{50}[1]	Top percentile based on NT_{50}[2]	NT_{80}[1]	VITROS S/CO[3]
W084520000915	60%	18,922	1%	2,313	736
W041020069696	20%	1,135	40%	541	345
W041120015179	20%	1,350	35%	454	506
**Pooled CCP**	**100%**	**3,003**	**20%**	**1,113**	**684**

Due to limited amount available of the plasma with the highest titer (W084520000915), a maximal amount of this highest-titer plasma was used and mixed with the 2 other units at a ratio of 60:20:20 in order to administer the maximum absolute amount of convalescent plasma-derived neutralizing antibodies to the animals**.**

[1]NT_{50} and NT_{80} titers determined by RVPN assay.

[2]Top percentile values of individual plasma units and the pooled CCP are based on 223 convalescent plasma samples with median NT_{50} titer of 784.

[3]Signal to cut-off ratio values on VITROS® Total Ig assay (which measures anti-spike IgG, IgM and IgA). A value of ≥1 is considered reactive.

bioRxiv preprint doi: https://doi.org/10.1101/2021.09.01.458520; this version posted September 1, 2021. The copyright holder for this preprint (which was not certified by peer review) is the author/funder. This article is a US Government work. It is not subject to copyright under 17 USC 105 and is also made available for use under a CC0 license.

Table S2. SARS-CoV-2 neutralizing and anti-spike antibodies in serum of macaques.

		Control group				Convalescent plasma group							
	Day / Animal nr	Co-1	Co-2	Co-3	Co-4	CCP-1	CCP-2	CCP-3	CCP-4	CCP-5	CCP-6	CPP-7	CCP-8
NT_{50}	0	< 40	< 40	< 40	< 40	< 40	< 40	< 40	< 40	< 40	< 40	< 40	< 40
	1	< 40	< 40	< 40	< 40	< 40	< 40	< 40	< 40	< 40	< 40	< 40	< 40
	2	< 40	< 40	< 40	< 40	111	159	152	195	80	100	169	90
	3	< 40	< 40	< 40	< 40	110	86	142	163	146	150	83	99
	5	< 40	< 40	< 40	< 40	159	85	126	132	60	81	< 40	61
	7	< 40	< 40	< 40	97	78	67	44	80	117	50	< 40	122
NT_{80}	0	< 40	< 40	< 40	< 40	< 40	< 40	< 40	< 40	< 40	< 40	< 40	< 40
	1	< 40	< 40	< 40	< 40	< 40	< 40	< 40	< 40	< 40	< 40	< 40	< 40
	2	< 40	< 40	< 40	< 40	< 40	116	< 40	64	41	< 40	48	< 40
	3	< 40	< 40	< 40	< 40	46	< 40	< 40	< 40	< 40	< 40	< 40	46
	5	< 40	< 40	< 40	< 40	131	< 40	< 40	< 40	< 40	< 40	< 40	< 40
	7	< 40	< 40	< 40	< 40	< 40	< 40	< 40	< 40	< 40	< 40	40	< 40
VITROS total Ig S/CO	0	0.05	0.05	0.03	0.04	0.09	0.04	0.09	0.04	0.02	0.03	0.04	0.03
	1	0.06	0.07	0.02	0.06	0.10	0.04	0.08	0.03	0.02	0.02	0.04	0.03
	2	0.07	0.06	0.03	0.08	3.83	55.29	20.35	24.52	91.83	41.23	22.61	17.23
	3	0.06	0.05	0.03	0.14	3.38	32.57	5.80	9.54	66.97	30.26	13.52	7.36
	5	0.10	0.11	0.05	0.17	3.26	19.84	3.65	6.36	32.54	14.16	7.61	6.47
	7	0.08	0.09	0.04	2.01	1.50	15.78	2.72	5.87	10.07	16.79	8.86	5.36

Animals were inoculated with SARS-CoV-2 on day 0, and control or convalescent plasma was infused on day 1. 50% and 80% neutralization titers (NT_{50} and NT_{80}) in serum were measured by a RVPN assay. Total Ig against spike protein was determined by the VITROS® assay. Green shading indicates values above the cut-off of the respective assay.

bioRxiv preprint doi: https://doi.org/10.1101/2021.09.01.458520; this version posted September 1, 2021. The copyright holder for this preprint (which was not certified by peer review) is the author/funder. This article is a US Government work. It is not subject to copyright under 17 USC 105 and is also made available for use under a CC0 license.

Table S3. Summary of radiological scoring.

Control plasma			Convalescent plasma		
Animal number	Day	Total Score	Animal number	Day	Total Score
Co-1	0	0	CCP-1	0	0
	1	0		1	0
	3	1		3	0
	5	1		5	0
	7	2		7	0
Co-2	0	0	CCP-2	0	0
	1	0		1	0
	3	0		3	1
	5	0		5	0
	7	0		7	0
Co-3	0	0	CCP-3	0	0
	1	0		1	0
	3	1		3	1
	5	0		5	0
	7	0		7	1
Co-4	0	0	CCP-4	0	0
	1	0		1	1
	3	0		3	1
	5	0		5	1
	7	0		7	0
			CCP-5	0	0
				1	0
				3	0
				5	0
				7	0
			CCP-6	0	0
				1	0
				3	0
				5	0
				7	0
			CCP-7	0	0
				1	0
				3	0
				5	0
				7	0
			CCP-8	0	0
				1	0
				3	2
				5	1
				7	0

All thorax radiographs were scored blinded by a veterinary radiologist, with scores of 0 to 3 assigned to each of the 7 lung lobes. For each time point, the total score of all lung lobes was tabulated. Thus, the maximum score per time point is 21.

bioRxiv preprint doi: https://doi.org/10.1101/2021.09.01.458520; this version posted September 1, 2021. The copyright holder for this preprint (which was not certified by peer review) is the author/funder. This article is a US Government work. It is not subject to copyright under 17 USC 105 and is also made available for use under a CC0 license.

Table S4. Animal demographics

Group	Animal ID	Sex	Age at time of inoculation (months)	Body weight at time of inoculation (kg)
Control plasma	Co-1	M	195	12.81
"	Co-2	F	169	9.31
"	Co-3	M	167	8.34
"	Co-4	F	110	6.09
Convalescent plasma	CCP-1	F	196	10.05
"	CCP-2	M	166	7.99
"	CCP-3	M	163	9.79
"	CCP-4	F	137	6.70
"	CCP-5	M	135	10.22
"	CCP-6	F	112	6.01
"	CCP-7	F	111	5.82
"	CCP-8	M	110	6.22

bioRxiv preprint doi: https://doi.org/10.1101/2021.09.01.458520; this version posted September 1, 2021. The copyright holder for this preprint (which was not certified by peer review) is the author/funder. This article is a US Government work. It is not subject to copyright under 17 USC 105 and is also made available for use under a CC0 license.

Table S5. Flow cytometry antibody and reagents.

S. No	Reagents	Source	Identifier
1.	AF488 anti-human Ki-67 (Clone B56)	BD Biosciences	Cat#558616
2.	AF700 anti-human CD14 (Clone MSE2)	BD Biosciences	Cat# 301822
3.	AF700 anti-human CD3 (Clone SP34-2)	BD Biosciences	Cat# 557917
4.	APC anti-human CD66 (Clone TET2)	Miltenyi Biotec	Order#130-118-539
5.	APC-Cy7 anti-human CD3 (Clone SP34-2)	BD Biosciences	Cat#557757
6.	APC-Cy7 anti-human CD20 (Clone 2H7)	BioLegend	Cat#302314
7.	APC-Cy7 anti-human live/dead	invitrogen	Ref#L34976A
8.	BV421 anti-human CD123 (Clone 7G3)	invitrogen	Ref#48-1238-42
9.	BV605 anti-human CD16 (Clone 3G8)	BioLegend	Cat#302040
10.	BV650 anti-human CD4 (Clone L200)	BD Biosciences	Cat# 563737
11.	BV786 anti-human HLA-DR (Clone L243)	BioLegend	Cat#307642
12.	BUV737 anti-human CD95 (Clone DX2)	BD Biosciences	Cat# 564710
13.	BUV805 anti-human CD8 (Clone SK1)	BD Biosciences	Cat#612889
14.	PECF594 anti-human CD28 (Clone CD28.2)	BioLegend	Cat# 302942
15.	PECy7 anti-human CD11c (Clone 3.9)	invitrogen	Ref#25-0116-42
16.	PECy7 anti-human PD1 (Clone EH12.2H8)	BioLegend	Cat# 329918
17.	FACS lyse	BD Biosciences	Cat#349202
18.	FoxP3/ Transcription Factor Staining Buffer set	invitrogen	Cat#00-5523
19.	Brilliant stain buffer	BD Biosciences	Cat#563794
